# microRNA Target Predictions across Seven *Drosophila* Species and Comparison to Mammalian Targets

**DOI:** 10.1371/journal.pcbi.0010013

**Published:** 2005-06-24

**Authors:** Dominic Grün, Yi-Lu Wang, David Langenberger, Kristin C Gunsalus, Nikolaus Rajewsky

**Affiliations:** Center for Comparative Functional Genomics, Department of Biology, New York University, New York, New York, United States of America; Lawrence Berkeley National Laboratory, United States of America

## Abstract

microRNAs are small noncoding genes that regulate the protein production of genes by binding to partially complementary sites in the mRNAs of targeted genes. Here, using our algorithm PicTar, we exploit cross-species comparisons to predict, on average, 54 targeted genes per microRNA above noise in *Drosophila melanogaster*. Analysis of the functional annotation of target genes furthermore suggests specific biological functions for many microRNAs. We also predict combinatorial targets for clustered microRNAs and find that some clustered microRNAs are likely to coordinately regulate target genes. Furthermore, we compare microRNA regulation between insects and vertebrates. We find that the widespread extent of gene regulation by microRNAs is comparable between flies and mammals but that certain microRNAs may function in clade-specific modes of gene regulation. One of these microRNAs *(miR-210)* is predicted to contribute to the regulation of fly oogenesis. We also list specific regulatory relationships that appear to be conserved between flies and mammals. Our findings provide the most extensive microRNA target predictions in *Drosophila* to date, suggest specific functional roles for most microRNAs, indicate the existence of coordinate gene regulation executed by clustered microRNAs, and shed light on the evolution of microRNA function across large evolutionary distances. All predictions are freely accessible at our searchable Web site http://pictar.bio.nyu.edu.

## Synopsis

MicroRNA genes are a recently discovered large class of small noncoding genes. These genes have been shown to regulate the expression of target genes by binding to partially complementary sites in the mRNAs of the targets. To understand microRNA function it is thus important to identify their targets. Here, the authors use their bioinformatic method, PicTar, and cross-species comparisons of several newly sequenced fly species to predict, genome wide, targets of microRNAs in *Drosophila*. They find that known fly microRNAs control at least 15% of all genes in *D. melanogaster*. They also show that genomic clusters of microRNAs are likely to coordinately regulate target genes. Analysis of the functional annotation of target genes furthermore suggests specific biological functions for many microRNAs. All predictions are freely accessible at http://pictar.bio.nyu.edu. Finally, Grün et al. compare the function of microRNAs across flies and mammals. They find that (a) the overall extent of microRNA gene regulation is comparable between both clades, (b) the number of targets for a conserved microRNA in flies correlates with the number of targets in mammals, (c) some conserved microRNAs may function in clade-specific modes of gene regulation, and (d) some specific microRNA–target regulatory relationships may be conserved between both clades.

## Introduction

Recently, it has been discovered that the genomes of animals contain hundreds of microRNA genes. These small noncoding genes are typically transcribed by RNA polymerase II, processed into hairpins, and exported into the cytoplasm, where they are cleaved by the central enzyme of the RNAi pathway, Dicer, to form single-stranded mature microRNAs [1,2]. In animals, mature microRNAs are thought to bind to partially complementary binding sites in the mRNAs of target genes and, by unknown mechanisms, to regulate their post-transcriptional expression. In all known cases microRNAs repress expression of protein-coding target genes, either by repressing translation while not affecting the mRNA concentration of the target, or potentially by directly inducing a decrease in target mRNA concentrations [3]. To understand the biological function of microRNAs it is therefore important to identify their targets. Since high-throughput experimental methods for microRNA target identification have not been published yet, computational methods that try to identify target sites based on their partial complementarity with microRNAs have become increasingly important [4–13]. In flies, the sensitivity of these methods was sufficient to predict roughly eight targets per microRNA above noise, although the true number of targets has been estimated to be much higher [14]. Cross-species comparisons, which allow for the identification of evolutionarily conserved and thus likely functional target sites, have proven very helpful to boost the sensitivity of microRNA target detection. Recently, three independent studies based on cross-species comparisons of eight vertebrates concluded that in vertebrates, microRNAs are predicted to regulate at least 20%–30% of all genes [8,13,15]. These findings are consistent with experimental results [3].

It has also been widely suggested that microRNAs, similarly to transcription factors, can act in combination (or cooperatively) by binding to the same mRNA in a concentration-dependent manner. Tissue specificity of gene expression could then be in part explained by a “microRNA code” [16] of tissue-specific expression of the *trans*-acting microRNAs. This idea is supported by experiments [17] and by results from computational approaches that have been used to search for target sites of different microRNAs in the same target mRNA [5,6,13]. In particular, a mammalian gene was predicted and experimentally shown to be coordinately regulated by several co-expressed microRNAs [13].

We used our microRNA-target-finding algorithm, PicTar [13], and cross-species comparisons of seven recently sequenced *Drosophila* species to predict and analyze microRNA targets in flies. Our underlying model for target site recognition and a comparison of these results to our previous predictions [9] is presented in the Discussion. We also computed predictions for common targets of clustered microRNAs, since recent experiments [18,19] have suggested that microRNA genes that reside in clusters spanning roughly 50 kbp of genomic DNA tend to be co-expressed. To shed light on the specific function of microRNAs, we analyzed the functional annotation for predicted target sets using Gene Ontology (GO) terms [20]. However, to arrive at a more global understanding of microRNA function we then asked whether the extent of microRNA targeting in flies is comparable to targeting in vertebrates, whether certain microRNA–mRNA regulatory relationships are conserved between both clades, and whether individual microRNAs could potentially play a role in clade-specific gene regulation.

## Results

### Genome-Wide Cross-Species Comparisons of Seven Fly Species Allow High-Specificity and High-Sensitivity microRNA Target Predictions

**Figure 1:**
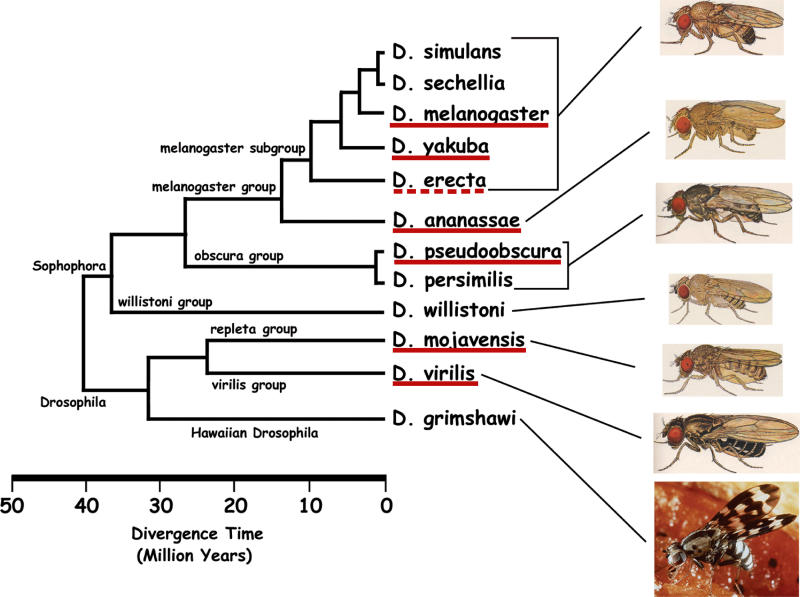
Phylogenetic Tree of 12 *Drosophila* Species Our datasets include 3′ UTRs for seven of these species: *D. melanogaster, D. yakuba, D. erecta, D. ananassae, D. pseudoobscura, D. virilis,* and *D.* *mojavensis*. Species underlined in solid red are present in set 1 and set 2. *D. erecta* (broken red line) is present only in set 2. Source: http://species.flybase.net/.

It has been widely demonstrated that the success of the computational identification of microRNA target sites can be significantly boosted by searching for target sites that are evolutionarily conserved, and therefore likely to be functional. Thus, we set out to make use of the very recent whole-genome sequencing of a number of fly species (Figure 1). The genomic sequence for eight of these species, which include members of the *melanogaster, obscura, repleta,* and *virilis* groups, have been already assembled *(D. melanogaster, D. simulans, D. yakuba, D. erecta, D. ananassae, D. pseudoobscura, D. virilis,* and *D.* *mojavensis).* We discarded the *D. simulans* assembly since it proved to contain large gaps. The estimated divergence time for these species ranges from a few million years to roughly 40 million years (Figure 1).

To identify evolutionarily conserved microRNA target sites in 3′ UTR sequences, it was critical to identify orthologous mRNAs. We experimented with two independently produced sets of genome-wide alignments of the eight species (see Materials and Methods). The first set of alignments (termed set 1), which does not contain sequence for *D. erecta,* was produced by the UCSC Genome database (http://genome.ucsc.edu/) and is based on pairwise alignments that were subsequently multiply aligned. The second set (termed set 2) came from true genome-wide multiple alignments (C. Dewey, MERCATOR, http://hanuman.math.berkeley.edu/~cdewey/mercator/) [21]. For both sets, we extracted multiple alignments of *D. melanogaster* 3′ UTRs using the *D.* *melanogaster* FlyBase annotation for 18,892 gene transcripts and obtained 3′ UTR alignments across all eight species for 13,465 transcripts (set 1) and 13,030 transcripts (set 2) (Table 1). We also defined sets of alignments by keeping only the longest 3′ UTR from all transcript variants for the same gene, resulting in approximately 9,800 alignments for each set (termed unique alignments). The coverage of genes is thus roughly comparable between both sets. Additionally we masked repeats in the unique alignments using the UCSC repeat masks for set 1 and using the Tandem Repeat Remover [22] following Rajewsky et al. [23] for set 2. The nucleotide space of the various alignment sets is listed in Table 2 and comprises for each set a total of 2.2–4.1 Mb per species for the repeat-masked unique alignments. Masking repeats thus removed substantial amounts of sequence (up to 22% per species).

**Table 1:**
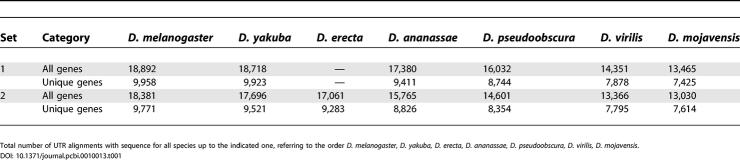
Statistics of the 3′ UTR Multiple Alignments

Total number of UTR alignments with sequence for all species up to the indicated one, referring to the order *D. melanogaster, D. yakuba, D. erecta, D. ananassae, D. pseudoobscura, D. virilis, D. mojavensis*.

**Table 2:**
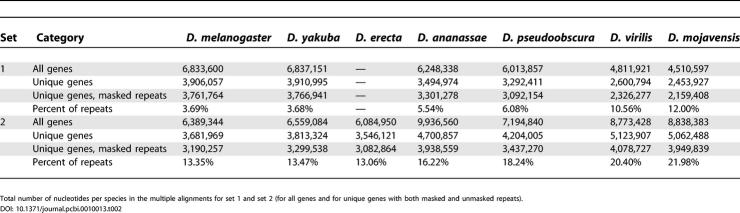
Number of Aligned 3′ UTR Nucleotides

Total number of nucleotides per species in the multiple alignments for set 1 and set 2 (for all genes and for unique genes with both masked and unmasked repeats).

To identify conserved microRNA targets, we used the algorithm PicTar [13]. The key component of PicTar is the notion of a “nucleus” (or “seed”), defined as a stretch of seven bases (starting at the first or second position from the 5′ end of the microRNA), with consecutive perfect Watson–Crick basepairings to the target site. A recent computational and experimental study [14] demonstrated that the presence of such a nucleus is necessary for a substantial fraction of all microRNA target sites in *Drosophila*. For the remaining sites the nucleus is imperfect and contains mismatches, bulges, or G:U basepairings. Experimental results have suggested that sites with imperfect nuclei seem to be functional only when compensated by additional binding of the 3′ end of the microRNA to the target site [14,17]. Input to PicTar consists of orthologous, aligned 3′ UTR sequences and a search set of one or several microRNAs. PicTar first determines candidate 3′ UTR alignments containing a minimal number of conserved perfect nuclei, termed anchor sites. The minimal number and the degree of conservation of anchor sites are defined by the user. Each candidate UTR is searched separately for sites with perfect and imperfect nuclei. Subsequently, imperfect sites are required to pass a free energy filter. This is currently set to maximally two-thirds of the free energy of the perfectly basepaired microRNA–mRNA duplex and thus removes the vast majority of sites with imperfect nuclei. Sites with a perfect nucleus may optionally be subject to a much milder free energy filtering step (depending on the settings). Finally PicTar computes a score (see Materials and Methods) reflecting the likelihood that a given UTR will be targeted by members of the search set based on a hidden Markov model.

**Figure 2:**
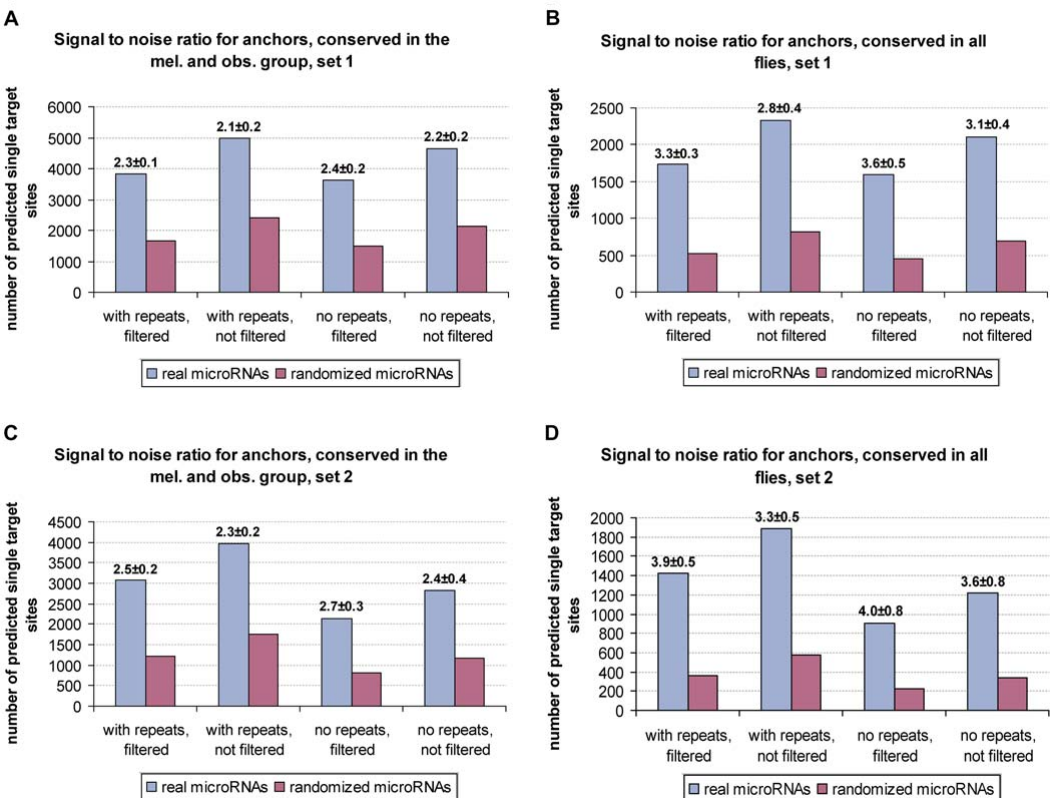
Signal-to-Noise Ratios of the PicTar Single Target Site Predictions For both set 1 and set 2 the predicted number of anchor sites for 46 unique microRNAs, conserved in all flies, and corresponding randomized microRNAs (averaged over five cohorts) and the respective signal-to-noise ratio (indicated above the bars) are shown with and without using free energy filtering of anchor sites for UTRs with either masked and unmasked repeats. (A) Predictions for set 1 with anchor sites conserved in the *melanogaster* and *obscura* groups. (B) Predictions for set 1 with anchor sites conserved in all flies. (C) Predictions for set 2 with anchor sites conserved in the *melanogaster* and *obscura* groups. (D) Predictions for set 2 with anchor sites conserved in all flies.

To estimate the extent of microRNA targeting in *Drosophila,* we used PicTar to count conserved putative target sites with perfect nuclei (anchors). The microRNAs used for these searches consisted of all currently known microRNAs that seemed to be conserved in all species under consideration (see Materials and Methods). To avoid counting target sites more than once, we represented all microRNA “families” that share identical nuclei by just one member of each family. The final set contained 46 microRNAs with unique nuclei conserved in all flies. As in our previous study [13], we recruited cohorts of randomized microRNA sequences to estimate the number of false positives (see Materials and Methods). Specifically, we computed all anchor sites (single conserved nuclei) for set 1 and set 2 with masked and unmasked repeats for real microRNAs, as well as for five sets of randomized cohorts in each case (Figure 2). A measure for the specificity is the signal-to-noise ratio, which is defined as the ratio of the number of anchor sites for real versus randomized microRNAs. In each case, we averaged the result over five cohorts and computed the mean and the standard deviation of the signal-to-noise ratio. We computed specificity and sensitivity, requiring different degrees of evolutionary conservation of anchor sites both with and without free energy filtering (Figure 2). Overall, we observed that using the free energy filter or masking repeats tends to enhance specificity with modest losses in sensitivity. We obtained higher signal-to-noise ratios with set 2, but a higher sensitivity with set 1. We also found that requiring different degrees of evolutionary conservation of anchor sites strongly affects sensitivity and specificity. More precisely, searching for anchor sites conserved between all flies (at various parameter settings) yielded a signal-to-noise ratio of 2.8–3.6 (set 1) and 3.3–4.0 (set 2). The sensitivity was, on average, 25–33 (set 1) and 15–29 (set 2) anchor sites per microRNA above noise. Anchor sites conserved in the *melanogaster* and *obscura* groups yielded signal-to-noise ratios of 2.1–2.4 (set 1) and 2.3–2.7 (set 2) with a sensitivity of 47–57 (set 1) and 29–40 (set 2) anchor sites per microRNA above noise (Figure 2).

**Figure 3:**
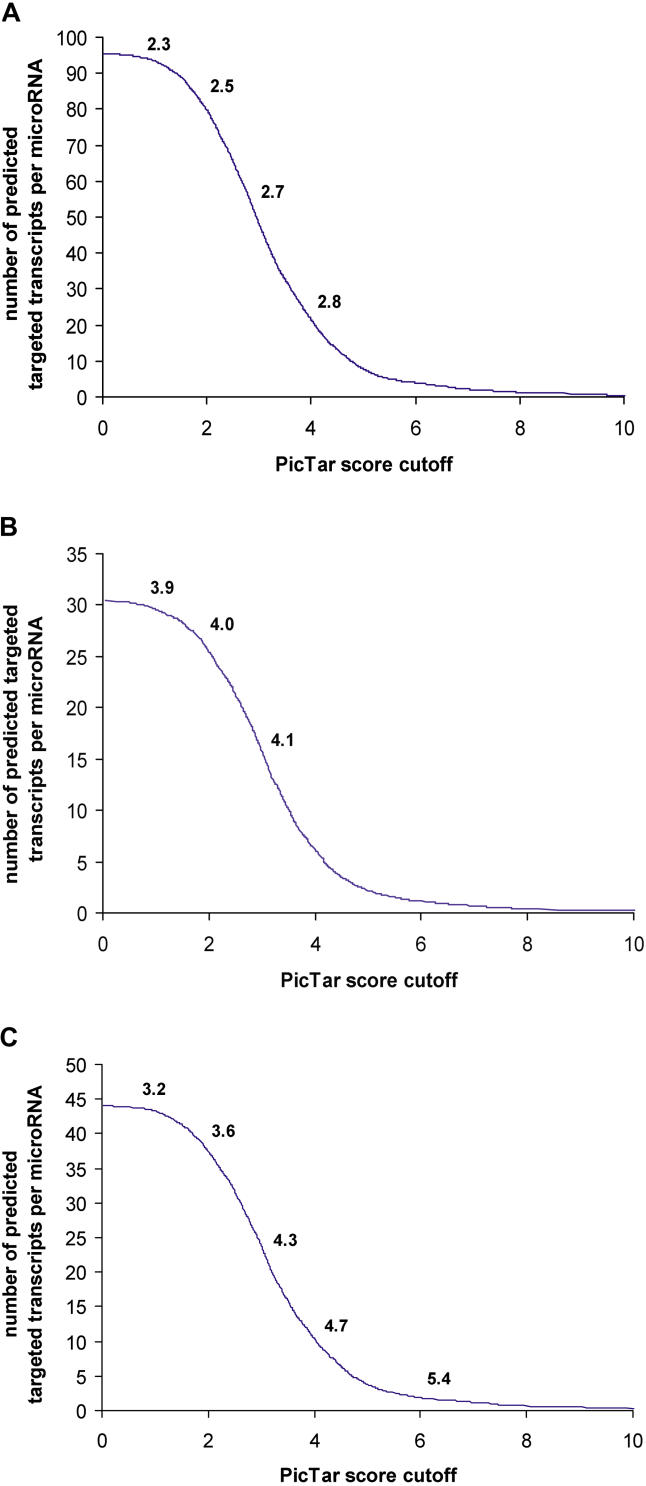
Sensitivity and Specificity as a Function of PicTar Score Shown is the average number of predicted targeted genes as a function of a PicTar score cutoff (discarding all target genes with a score below this cutoff) for three different PicTar settings (S1–S3; see Materials and Methods): (A) high-sensitivity setting (S1), (B) high-specificity setting (S2), and (C) medium sensitivity/medium specificity setting (S3). The signal-to-noise ratio also depends on the score cutoff and is indicated above the curve for certain cutoff values. All predictions for all settings can be accessed on the PicTar Web server (not filtered by score cutoffs).

Based on these results we defined three settings, termed S1, S2, and S3 (see Materials and Methods) that allowed us to adjust the trade-off between sensitivity and specificity, and to generate predictions of high sensitivity, high specificity, and medium specificity/sensitivity, respectively. For each of the settings S1–S3 we recorded the specificity and the number of targeted transcripts as a function of the PicTar score cutoff, i.e., discarding all predictions with a score lower than a given threshold (Figure 3). We found that high-scoring transcripts tended to have a significantly improved specificity. For example, when using setting S3 the signal-to-noise ratio can be improved by a factor of 1.7 while retaining a sizeable number of predicted transcripts per microRNA. The positive correlation between specificity and PicTar score is consistent with our observation that some non-anchor sites make a contribution to the score. These sites appear to be “scattered”, i.e., are present only in some species or are not found in all species at the same position in the alignment. We experimented with relaxing our anchor site definition to include cases where a perfect nucleus is found in all species under consideration but not necessarily at overlapping positions in the alignments. The signal-to-noise ratio decreased in all settings S1–S3 (for example for S3 from 3.3 to 2.6), with no significant gain in sensitivity. We thus concluded that many scattered sites could be functional but should be scored only when they occur in conjunction with anchor sites, as implemented in the PicTar algorithm.

**Figure 4:**
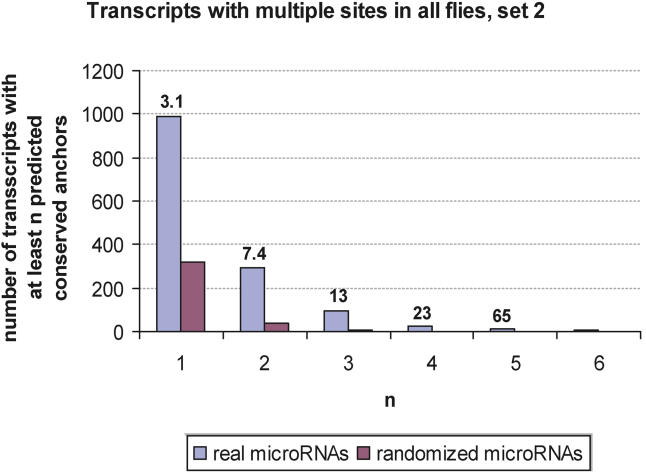
Specificity of PicTar Predictions of Genes with Multiple Putative Target Sites Number of unique genes as a function of the minimal number of anchor sites for 46 unique, conserved microRNAs and for randomized microRNAs (averaged over five cohorts). The ratio of these numbers, reflecting the specificity, is indicated above each bar.

Previous analyses of microRNA targeting in vertebrates [6,8,13,15] and flies [5,14] suggested that a substantial fraction (10%–30%) of all protein-coding genes in both clades are regulated by microRNAs. Using settings S3 (or S2), we found that 15% (13%) of all annotated roughly 10,000 unique *melanogaster* 3′ UTR transcripts (corresponding to approximately 10,000 genes) have at least one anchor site that is conserved in all seven fly species at a signal-to-noise ratio of about three (four). Thus, with settings S3 or S2, roughly 10% of all transcripts are predicted to be targeted by microRNAs above noise in all flies. To estimate how many genes could be regulated by more than one microRNA, we counted all transcripts with at least two anchor sites. Applying the high-specificity setting S2, we found that searching for multiply targeted transcripts further enhances the specificity to a significant degree (Figure 4). For example, we found seven times as many targeted transcripts with at least two anchor sites for real microRNAs compared to randomized microRNAs. With settings S2 and S3, we predicted that 30% of all targeted transcripts have more than one anchor site. Finally, for our high-sensitivity setting S1 we found that 27% of all transcripts have at least one anchor site at a single-site signal-to-noise ratio of approximately 2.2. Of these, 40% are found to have at least two anchor sites.

In summary, based on our high-sensitivity setting, we predicted that at least 15% of all *D.* *melanogaster* genes with currently annotated 3′ UTR sequences are regulated by at least one known microRNA, and that at least one-fifth of these *Drosophila* microRNA targets could be subject to coordinate control by two or more microRNAs from different microRNA families (above noise). We provide ranked PicTar target predictions for all conserved microRNAs, all FlyBase transcripts, and settings S1–S3 at our searchable Web site (http://pictar.bio.nyu.edu). The results, linked to various other public databases, can be queried for genes of interest or microRNAs of interest.

### Recovery of Experimentally Validated microRNA Targets in *Drosophila*

We have previously shown that PicTar has an excellent recovery rate of validated *Caenorhabditis elegans* microRNA targets [13]. To analyze the recovery of experimentally validated targets in *Drosophila,* we collected 19 microRNA–target regulatory relationships from the literature [4,12,24]. The overlap with PicTar predictions across settings S1–S3 is summarized in Table 3. The apoptosis gene* hid/wrinkled* is targeted by the microRNA* bantam* [24]. For all settings S1–S3, *hid* is the top-scoring *bantam* target (PicTar score of 17.3) and has five anchor sites conserved in all flies. Notably, *hid* targeted by *bantam* has the second highest PicTar score within all our target predictions. The only gene with a higher score (40.5) is *nerfin-1,* which contains two anchor sites for *miR-286* (or equivalently *miR-279*) conserved in all flies, and many additional sites for the same microRNA (see Discussion).

**Table 3:**
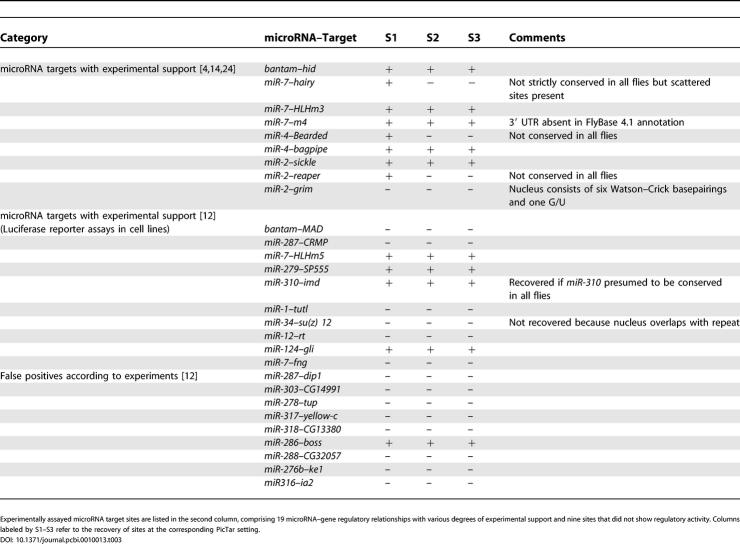
Recovery of Published *Drosophila* microRNA Targets with Experimental Support

Experimentally assayed microRNA target sites are listed in the second column, comprising 19 microRNA–gene regulatory relationships with various degrees of experimental support and nine sites that did not show regulatory activity. Columns labeled by S1–S3 refer to the recovery of sites at the corresponding PicTar setting.

The *Notch* signaling gene *hairy* was recently predicted [4,9] and validated as a target of *miR-7* with a single binding site [4]. PicTar found a *miR-7* anchor site conserved in all flies of the *melanogaster* and *obscura* groups, whereas the site in *D. virilis* appears to be slightly shifted upstream. Hence, this target is recovered with setting S1 but not with settings S2 and S3. There is experimental evidence that *miR-7* also targets *HLHm3* and *E(spl)m4,* two genes that are located in the *E(spl)* complex [4]. For *HLHm3,* PicTar predicts one *miR-7* target site conserved in all flies (with all settings). The gene *E(spl)m4* did not have an annotated 3′ UTR but was recovered after adding the likely 3′ UTR sequence to our dataset [4]. Another gene of the *E(spl)* complex, *HLHm5,* is the highest ranking target gene of *miR-7* when searching for targets conserved in all flies (with setting S2; rank 2 with setting S3). Target predictions at a reduced level of conservation (setting S1) also yield *HLHm5* as the top-ranking *miR-7* target. The *Notch* gene *Bearded* is recovered as a target of *miR-4* (or *miR-79,* equivalently). With setting S1 we found three conserved sites in its 3′ UTR. These so called *Bearded* boxes have been shown to mediate repression of a reporter gene with a *Bearded* 3′ UTR in vivo [25]. This gene is again very high scoring (15.6) and ranks second in the list of* miR-4* target predictions (setting S1). This target is not recovered with the other settings, because the alignments of this gene do not contain sequence for *D. mojavensis* and *D. virilis.* The same microRNA is thought to repress* bagpipe* [14], which ranks second in the list of* miR-4* target predictions (S3).

The proapoptotic genes *reaper, grim,* and *sickle* are validated targets of the *miR-2* family [4]. For *sickle* we found one conserved site in all flies for *miR-2, miR-13,* and *miR-6,* which share the same nucleus. For *reaper,* we recovered one site for the same microRNAs in the *melanogaster* and *obscura* group with setting S1, while the other settings failed to identify this target because of missing sequence for this gene in *D. mojavensis.* *grim* is the only target of this group not recovered by PicTar, because it has only a 6mer nucleus for *miR-2*.

A recent algorithm for the prediction of microRNA targets did not rely on evolutionary information, but incorporated the 3′ UTR secondary structure to compute putative microRNA targets [12]. Some of the high-scoring predictions could then be supported by luciferase reporter constructs in cell lines. We recovered four targets from this list *(miR-7/HLHm5, miR-279/SP555, miR-124/Gli,* and *miR-310/imd)* but failed to locate conserved nuclei for the other six targets (see comments in Table 3). Strikingly, out of nine computationally predicted targets that were experimentally assayed but did not show any repression activity (likely false positives) [12], we only predicted one microRNA–target regulatory relationship *(miR-286/boss).*

In summary, PicTar recovered 8/9 (89%) of all known targets with experimental in vivo evidence and 4/10 (40%) of targets with other experimental support with setting S1, i.e., requiring conservation of anchor sites only in flies of the *melanogaster* and *obscura* groups. Only three of all targets with experimental support were lost when requiring conservation between all fly species and thus were not recovered with settings S2 and S3.

### Some Clustered microRNAs Are Likely to Coordinately Regulate Gene Expression

Expression assays have shown that microRNA genes that are located in the same genomic region within 50 kb of each other are often co-expressed [18,19], suggesting the possibility that they may coordinately regulate common target genes. In *D. melanogaster*, we identified seven clusters within 50-kb regions that contained precursors of at least two conserved microRNAs from different families. To identify common targets of clustered microRNAs in flies, we used PicTar to predict coordinate targets for each of these microRNA clusters (available on the PicTar server). Table 4 gives an overview of all clusters, their location in the *Drosophila* genome, the abundance of targeted transcripts, and, whenever all microRNA genes of a given cluster are located in an intron of another gene, the identifier of this gene. To evaluate whether clustered miRNAs target the same gene more often than expected by chance, we considered all 1,128 pairwise combinations of all 48 unique conserved microRNAs. While pairs of microRNAs from the same cluster make up only 2.1% of these pairs, 132 genes contained at least one anchor site for each microRNA of these clustered pairs (using setting S1), or 12% of the 1,104 genes that contain at least two different anchor sites for any combination of these 48 microRNAs. Thus, some pairs of microRNAs from clusters are likely to coordinately regulate a significantly higher proportion of genes (12%) than expected (2.1%). Furthermore, the number of target genes predicted for pairs of clustered microRNAs is twice the number expected from randomly drawn sets of 24 pairs among the 48 conserved microRNAs, which is significant by three standard deviations (see Materials and Methods). These findings support the hypothesis of coordinate control executed by clustered microRNAs.

**Table 4:**
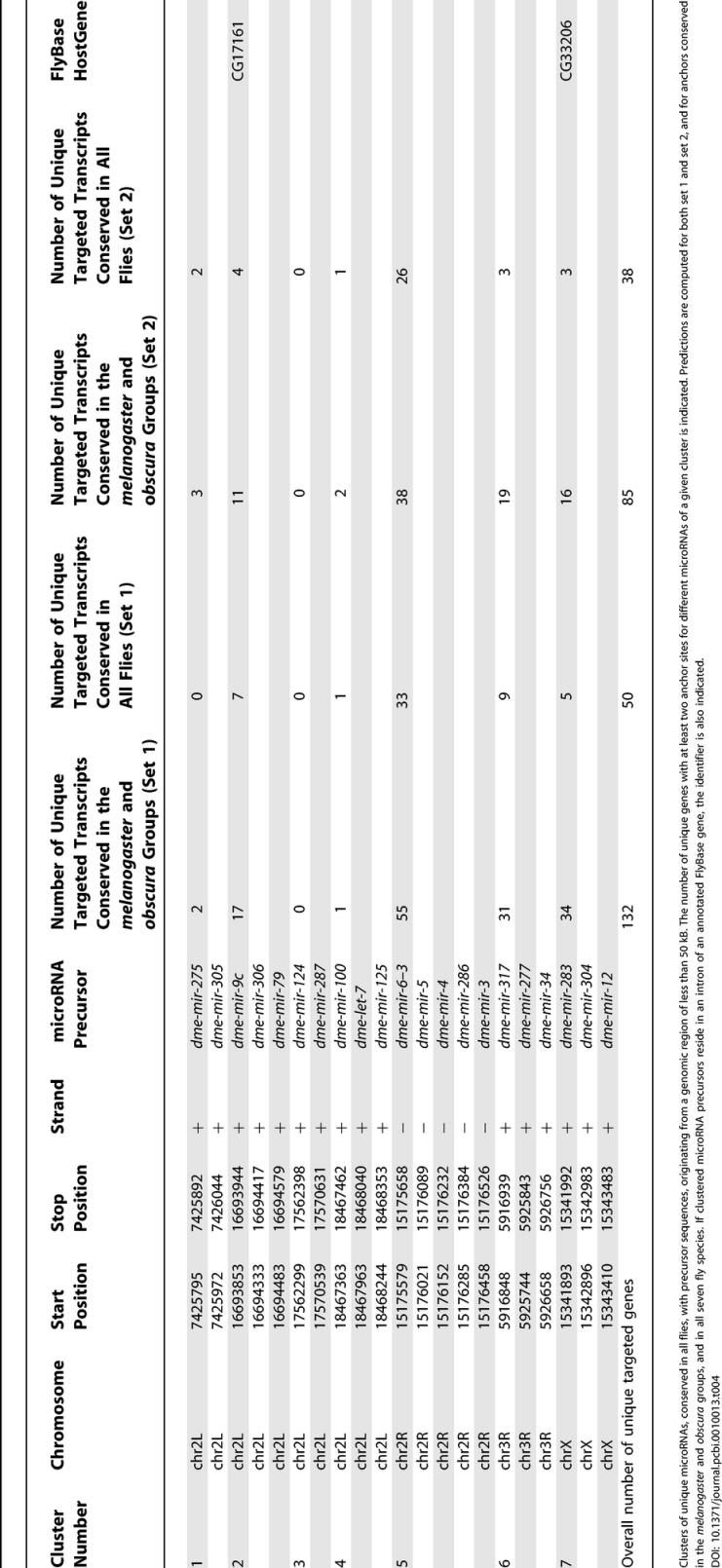
Clusters of microRNAs and Their Number of Predicted Target Genes

Clusters of unique microRNAs, conserved in all flies, with precursor sequences, originating from a genomic region of less than 50 kB. The number of unique genes with at least two anchor sites for different microRNAs of a given cluster is indicated. Predictions are computed for both set 1 and set 2, and for anchors conserved in the *melanogaster* and *obscura* groups, and in all seven fly species. If clustered microRNA precursors reside in an intron of an annotated FlyBase gene, the identifier is also indicated.

### Biological and Molecular Classification of Predicted microRNA Targets

**Figure 5:**
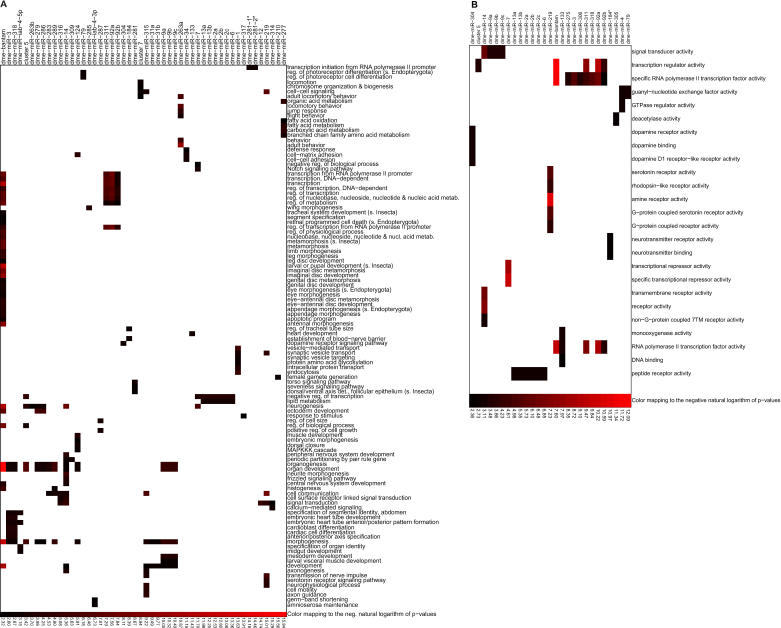
Significant GO Terms among the Predicted Target Genes of All Single microRNAs and Clusters of Co-Expressed microRNAs Significantly enriched GO terms for (A) “biological processes” and (B) “molecular function” ontologies. Shown are GO terms with *p*-values smaller than 0.1, corrected for multiple testing. Hierarchical clustering was performed separately for GO terms and microRNAs (see Materials and Methods).

To gain insight into the function of *Drosophila* microRNAs, we used GeneMerge [26] to analyze the over-representation of specific GO terms [20] in the functional annotation of genes predicted to be targeted by a particular microRNA versus a background gene set (see Materials and Methods). To avoid potentially spurious statistical significances, we chose not to use all genes as the background, but constructed a background set comprising all predicted targets for both real and randomized microRNAs. From the “biological process” ontology, a total of 112 significantly over-represented GO terms were identified; 70% of the gene sets targeted individually by conserved microRNAs and two sets of combinatorial target predictions for microRNA clusters contained at least one over-represented GO term (Figure 5A). For the “molecular function” ontology, a total of 25 significantly over-represented GO categories were obtained among 36% of all individual microRNA target gene sets and one set of microRNA cluster targets (Figure 5B). Consistent with previous estimates [1,2], our data indicate that microRNAs regulate a large variety of genes in many different biological processes. Globally prominent GO terms were morphogenesis, organogenesis, development (including embryonic development, and anterior/posterior and dorsal/ventral axis specification), neurogenesis, signal transduction (including Notch, Torso, Sevenless, and Frizzled signaling), and transcriptional regulation. Our overall overlap with another GO analysis for fly microRNA targets in a recent study was marginal, very likely because of not only the differences in approaches for identifying over-represented GO terms, but also the different nature of target site predictions made by PicTar and the published miRanda algorithm [5].

Our data were consistent with and extended results from a recent study that used GO functional analysis to predict microRNA target genes [4], in which *miR-7* was predicted to be active in Notch signaling and *miR-277* in valine, leucine, and isoleucine degradation. For *miR-277,* we recovered all nine predicted targets and found five additional genes (CG3267, CG4389, CG4600, CG6638, and CG8778) at *p* < 10^−7^. Targets of *miR-7* predicted by PicTar included many Notch pathway genes as well as targets of Notch signaling, including *E(spl)m5, Tom, Bob, E(spl)mγ, Bearded, E(spl)m3,* and *E(spl)m4,* most of which were very high scoring (using setting S1). Furthermore, many targets of Notch signaling were also predicted as targets of the Bearded-box microRNAs *miR-4* and *miR-79 (E(spl)m5, Bearded, E(spl)mγ,* and *Tom)* and of the K-box microRNAs *miR-2* and *miR-11* *(E(spl)m5, E(spl)m2, E(spl)mδ,* and *E(spl)m3),* consistent with previous observations [27]. Other known Notch targets would have been included in PicTar's target lists if their 3′ UTRs were annotated in the current FlyBase release (data not shown). We note that the majority of Notch targets predicted by PicTar would not have been predicted if stringent free energy filtering were applied for predicted microRNA–target duplexes with perfect nuclei.

### Comparison of microRNA Targets between Flies and Vertebrates

**Figure 6:**
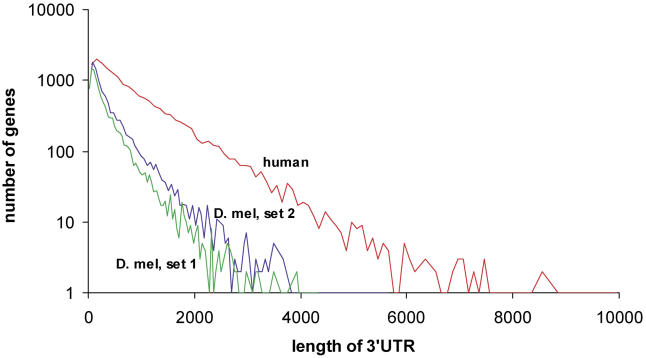
Lengths Distribution of 3′ UTRs in Human and *D. melanogaster* Data for set 1 and set 2 on a logarithmic scale. The distribution decays exponentially with increasing length in human much slower than in *D. melanogaster*. The average 3′ UTR lengths in human and *D. melanogaster* are approximately 900 and approximately 400 nucleotides, respectively.

**Figure 7:**
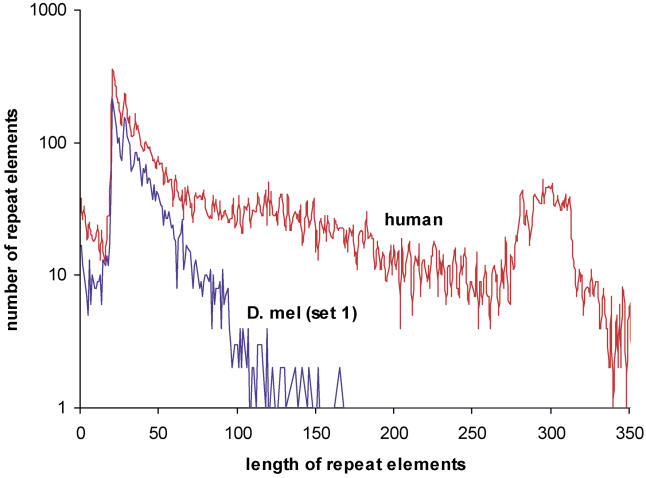
Length Distribution of Repeat Elements in 3′ UTRs of Human and *D. melanogaster* Data for set 1 on a logarithmic scale. The distribution peaks strongly for both species at a length of 11 nucleotides and decays exponentially for longer repeat elements in *D. melanogaster*. Up to a length of roughly 50 nucleotides, both distributions are very similar, while for longer elements the distribution for human no longer decays exponentially, but has a broad tail with another significant peak at a length of approximately 300 nucleotides.

Previously, we applied PicTar to exhaustively search 3′ UTR alignments of eight vertebrates (human, chimpanzee, mouse, rat, dog, chicken, pufferfish, and zebrafish) for microRNA target sites [13]. To compare the extent of microRNA targeting in flies and vertebrates, we first compared length, repeat content, and conservation of 3′ UTRs between both clades, using our datasets derived from the UCSC database for consistency. We focused on the comparison of 3′ UTRs between *D. melanogaster* and human since 3′ UTRs from these species were extracted based on annotated transcripts. We found that the length distribution of 3′ UTRs and the distribution of repeats within them are very similar between all mammals and between all flies, respectively, so comparisons between human and *D. melanogaster* UTRs should reveal essential differences between the two clades. We found a much broader distribution of 3′ UTR lengths in mammals than in flies, yielding on average approximately 900 nucleotides per 3′ UTR for human and approximately 400 nucleotides per 3′ UTR in* D. melanogaster* (Figure 6), consistent with previous results [28]. Examining the contribution of repeat elements, we found that repeats constitute 11% of all human 3′ UTR sequences compared with 4% in *D. melanogaster* (Table 5). Interestingly, for short repeats (up to about 50 nucleotides), the length distribution in* D. melanogaster* and human is similar (Figure 7). For longer elements the distribution in flies continues to decay exponentially with the same slope, whereas the human distribution displays a broad tail with another significant peak centered around approximately 300 nucleotides. To analyze 3′ UTR conservation, we counted all 7mers that appeared to be perfectly conserved in each 3′ UTR multiple alignment and divided these counts by the length of the 3′ UTR sequence. We found that the probability of a nucleotide to reside in a conserved 7mer is comparable between vertebrate alignments (including human, chimp, mouse, rat, dog, and chicken) and alignments covering all fly species in our dataset (0.02 and 0.03, respectively). Similarly, 3′ UTR conservation is comparable between mammals and flies in the *melanogaster* and *obscura* groups (0.06 and 0.08, respectively). The contribution of repeat elements to conserved 7mers is substantially different in vertebrates and flies (Table 6). Masking repeats reduced the number of bases in conserved 7mers by about 1% in vertebrates and about 10% in flies. Thus, repeats in 3′ UTRs appear to be much better conserved in flies than in vertebrates and thus may be of functional importance in flies.

**Table 5:**
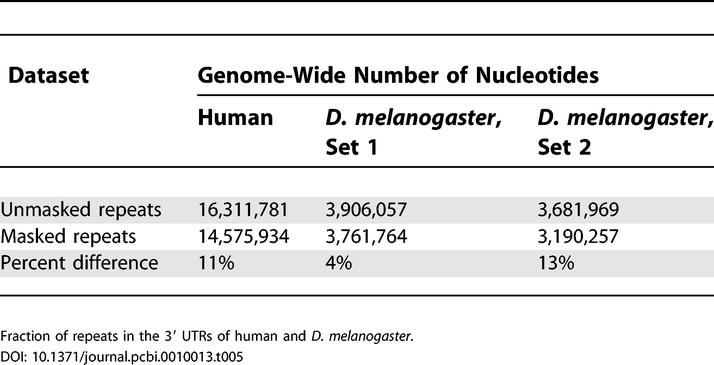
Repeat Elements in 3′ UTRs of Human and *D. melanogaster*

Fraction of repeats in the 3′ UTRs of human and *D. melanogaster.*

**Table 6:**
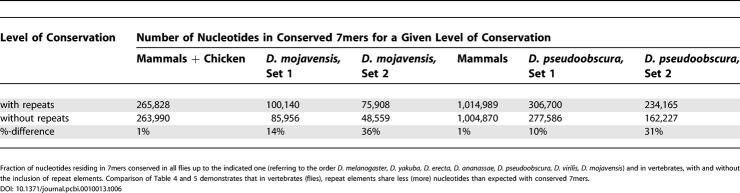
Conservation of 7mers in 3′ UTRs of Vertebrates and Flies

Fraction of nucleotides residing in 7mers conserved in all flies up to the indicated one (referring to the order *D. melanogaster, D. yakuba, D. erecta, D. ananassae, D. pseudoobscura, D. virilis, D. mojavensis*) and in vertebrates, with and without the inclusion of repeat elements. Comparison of Table 4 and 5 demonstrates that in vertebrates (flies), repeat elements share less (more) nucleotides than expected with conserved 7mers.

**Figure 8:**
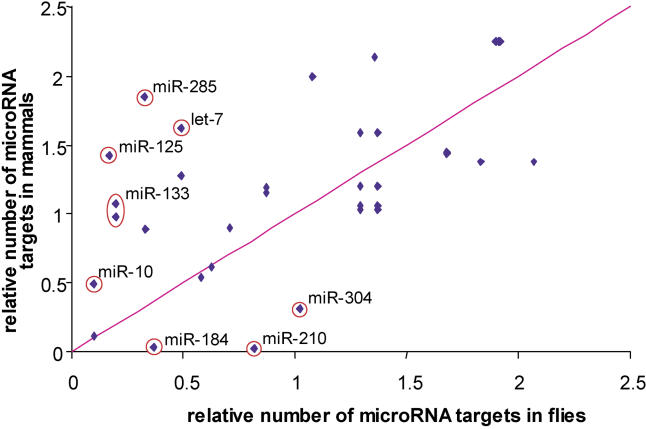
Number of Predicted Target Genes for Homologous microRNAs between Mammals and Flies Scatter plot for relative numbers of targeted genes predicted for homologous microRNAs in mammals and flies. The ratio of the number of predicted target genes of a microRNA and the average number of putative targeted genes per microRNA are plotted in mammals (*y*-axis) versus flies (*x*-axis). Conservation in flies included the *melanogaster* and *obscura* groups. Outliers (with a ratio of relative numbers of predicted target genes larger than 3.0 or smaller then 0.33) are circled. The microRNA identifiers refer to microRNAs annotated in *D. melanogaster*.

The extent of microRNA regulation seems roughly comparable between mammals and flies overall, with several interesting clade-specific differences. In vertebrates, we and others [6,8] found that roughly 30% of all genes may be regulated by microRNAs. This is twice the number we found in flies (15%), but this could be explained by the smaller number of known microRNAs in flies and other reasons (see Discussion). More interestingly, we checked whether individual microRNAs appeared to target similar or significantly different numbers of genes in mammals versus flies, since such differences could be indicative of clade-specific changes in microRNA function. To retain a reasonable sensitivity in target predictions for this analysis, we used human, chimp, mouse, rat, and dog for target predictions in mammals and the *melanogaster* and *obscura* groups for predictions in flies. We defined a set of 48 homologous microRNAs in mammals and flies (see Materials and Methods) and computed the average number of microRNA targets in both clades. We then calculated the ratio of predicted targets per microRNA to the average separately for each clade (Table 7). A scatter plot of these ratios (Figure 8) demonstrates a correlation between the numbers of targeted genes for homologous microRNAs in mammals and flies. However, certain microRNAs appear to have a significantly higher number of target genes in either humans *(miR-10, miR-133, miR-125, let-7,* and *miR-285)* or flies *(miR-184* and* miR-210).* For example, for *let-7* we found 1.64 as many target genes as expected on average in mammals, but only around 50% of the average expected number in flies. It is impossible to determine from this analysis whether microRNAs have acquired more targets in one clade or lost targets in the other, but it is striking that both human homologs of the fly microRNAs *miR-184* and *miR-210* are expressed at low abundance across many human tissues, while the homologs of *miR-10, miR-133, miR-125, let-7,* and *miR-285* are expressed overall at much higher levels [19]. We stress that the human homologs of *miR-10* and *miR-133* have average or below average numbers of predicted targets in human. Our data indicate that the above seven microRNAs may function in clade-specific modes of gene regulation.

**Table 7:**
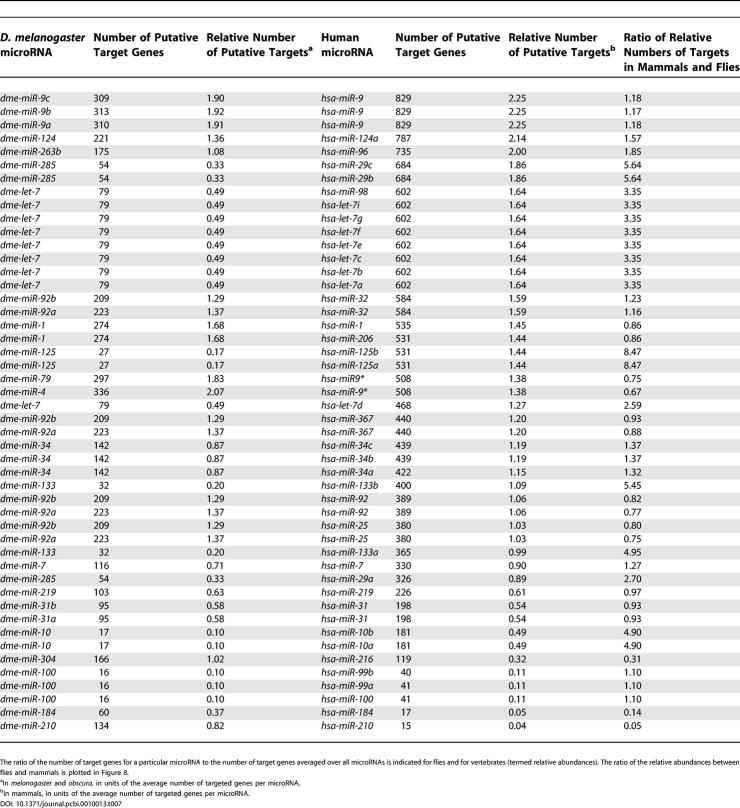
Homologous microRNAs between Mammals and Flies of the *melanogaster* and *obscura* Groups and Their Respective Number of Target Genes

The ratio of the number of target genes for a particular microRNA to the number of target genes averaged over all microRNAs is indicated for flies and for vertebrates (termed relative abundances). The ratio of the relative abundances between flies and mammals is plotted in Figure 8.

^a^In *melanogaster* and *obscura,* in units of the average number of targeted genes per microRNA.

^b^In mammals, in units of the average number of targeted genes per microRNA.

Finally, we computed which regulatory microRNA–mRNA relationships seemed to be conserved between flies and mammals (see Materials and Methods). From all 8,136 homologous human–*D*. *melanogaster* gene pairs in our dataset, 50 unique gene pairs were predicted to be targeted by homologous microRNAs (listed in Table S1). These 50 pairs comprise approximately 60 microRNA–mRNA regulatory relationships. Although these numbers are small, stringent permutation tests indicated that the result was marginally significant (1.7 standard deviations) (see Materials and Methods). Perhaps not surprisingly, almost half of the 50 *D. melanogaster* genes belong to the GO category “development,” and “histogenesis” is assigned to 13 of these 24 genes. Both results are significant (see Materials and Methods).

## Discussion

### The Extent of Post-Transcriptional Gene Regulation in *Drosophila* Mediated by microRNAs

The sequencing of the genomes of several *Drosophila* species proved to be an invaluable resource for the analysis of microRNA targets in flies. Cross-species comparisons allowed us to arrive at significantly enhanced sensitivity and specificity for microRNA target predictions in comparison with recent approaches. For example, previous studies have predicted on average eight target genes per microRNA (see [14] and references therein), while our data allow us (with high-sensitivity setting S1) to predict 54 target genes per microRNA above noise in *D. melanogaster*. Requiring conservation in all flies, we still predict on average more than 23 and 30 target genes per microRNA, for settings S2 and S3 respectively, at a strongly enhanced signal-to-noise ratio.

Based on our target predictions, we found that currently known microRNAs are expected to regulate a large fraction of all *D. melanogaster* genes (15%). This number is almost certainly an underestimate, since (a) the annotation of 3′ UTRs is incomplete, (b) the genomic sequences of several fly species still contain large gaps, and (c) it is expected that many more microRNAs in fly remain to be discovered. Indeed, using an approach analogous to that of a recent comparative study of mammals [15], we analyzed fly 3′ UTRs across all seven species and found strong evidence for the existence of a substantial number of yet undiscovered fly microRNA genes (N. Rajewsky, unpublished data).

The number of targets per microRNA we predicted is consistent with recent estimates of the true number of microRNA targets by Brennecke et al. [14]. In that study, the authors analyzed the statistical significance of conserved 8mer nuclei and conserved 7mer nuclei and concluded that the vast majority of computationally detectable target sites possessed at least one conserved 7mer nucleus. Our method is similar to this approach, but differs in the larger number of species included in our conservation analysis. Requiring similar levels of sequence conservation yields roughly comparable numbers of target genes per microRNA for both methods. In a number of cases in our dataset, gaps in the assemblies artificially decrease the number of predicted targets. On the other hand, using all seven *Drosophila* species allowed us to almost double the signal-to-noise ratio. In the future, further completion of the assemblies of the *Drosophila* genomes will almost certainly boost the number of PicTar predictions.

### Comparison to Our Previous Algorithm

Previously, we had published an algorithm for microRNA target identification and used it to predict microRNA targets within a set of central developmental genes involved in the body patterning of *Drosophila* [9]. In our model for target site recognition, we had introduced the notion of the nucleus as a stretch of perfect Watson–Crick basepairings between the microRNA and the target site and had shown that the nucleus (a) is typically 6–8 bases long, (b) is the central component of the specificity of target recognition, and (c) may serve as a nucleation site to allow a rapid zip up of the nucleus region of the microRNA–mRNA duplex [9]. This model for target site recognition explicitly proposed an explanation for the physical basis of target site recognition that combined kinetic and thermodynamic components. A recent experimental paper supports this idea [29]. We had also observed that the position of the nucleus within the microRNA is oftentimes conserved and at the 5′ end, indicating that the same *cis*-regulatory motif may be used to coordinate the action of a microRNA across different genes. We compared our previously predicted microRNA–mRNA regulatory relationships to our current PicTar predictions. We found that out of all cases where genes were present in both datasets, 11 out of 30 previous predicted sites were precisely recovered by PicTar. A number of the predictions are not recovered by PicTar because our previous algorithm did not restrict the nucleus to the 5′ end of the microRNA.

### Future PicTar Improvements

The highest scoring gene from all single microRNA target site predictions was* nerfin-1,* with two anchor sites for* miR-286* conserved in all flies and many additional, non-aligned sites present in all flies. Errors or ambiguities in the alignment can oftentimes explain the presence of these “scattered” sites. Additionally, compensatory mutations could lead to non-aligned and yet functionally conserved target sites in a 3′ UTR. At present, PicTar scores these scattered sites in the same way as it scores conserved sites, as long as both of them occur in the same UTR. Future refinements of the algorithm should explore (a) explicit evolutionary models for the evolution of 3′ UTR sequences and microRNA target sites, (b) improved probabilistic scoring for sites with imperfect nuclei [14], (c) the incorporation of secondary structure information [12], (d) incorporation of mRNA expression levels (e.g., from microarray experiments), and (e) expression levels of microRNAs.

Our data indicated that some clustered microRNAs are likely to coordinately regulate target genes. In addition, it has been shown that clustered microRNAs are likely to be co-expressed. Using multiple co-expressed microRNAs to coordinately regulate target genes could be an efficient way to increase the specificity of target gene regulation, and may also enhance the robustness of target gene expression levels against fluctuations in individual microRNA concentrations. We note that our data only suggest that clustered microRNAs are more likely to coordinately regulate target genes by coordinate binding to their 3′ UTRs than non-clustered microRNAs. Many microRNAs that reside in clusters also seem to target genes without additional binding sites for microRNAs in the same cluster. Conversely, there appear to be many possibilities for microRNAs from different clusters to coordinately bind the same target genes.

### The Evolution of microRNA Function across Large Evolutionary Distances

microRNAs offer the exciting possibility to study the evolution of *trans*-acting regulatory genes together with the evolution of their *cis*-regulatory target sites using computational methods. In this study, we have only touched upon this problem by comparing the estimated number of targeted genes per microRNA in one clade to the predicted number of targets for the homologous microRNA in another clade, which, by our definition of homology, is likely to bind to the same *cis*-regulatory sites. We caution that our definition of homology would also refer to microRNAs that may have evolved independently in one or both clades. However, our comparison yielded a nontrivial correlation between the numbers of targeted genes per microRNA in flies and vertebrates, indicating that the relative number of microRNA targets per microRNA tends to be conserved over very large evolutionary distances. In contrast, only a relatively modest number of specific microRNA–mRNA regulatory relationships seemed to be conserved between both clades. This scenario hints at conservation of global “network” features of gene regulation mediated by microRNAs while implicating microRNAs in an extensive rewiring of post-transcriptional gene regulation during organismal evolution.

It was striking that some microRNAs (including *let-7*) that are likely to have a large number of target genes in vertebrates seem to have a strongly reduced relative number of targets in flies, and vice versa. We singled out three microRNAs *(miR-184, miR-304,* and *miR-210)* with a drastically enhanced relative number of targets in flies compared to vertebrates. Our GO term analysis for microRNA targets revealed that one of them *(miR-210)* had over 70 predicted target genes, which as a group were significantly enriched (*p* < 0.03 after correcting for multiple testing) for 11 genes with the GO annotation “female gamete generation” (see Figure 5A). These 11 predicted *miR-210* targets are *cut, egghead, germ cell-less, gurken, lozenge, par-1, Ras oncogene at 85D, rhomboid-4, RNA-binding protein 9, singed,* and* slalom.* Most of these genes are evolutionarily conserved and have a known role in *Drosophila* oogenesis, either in development and patterning of the oocyte or in differentiation of the somatic follicle cells that surround the developing egg chamber, and seven of the 11 are implicated in developmentally critical signaling pathways involving receptor tyrosine kinases, Notch, wingless, or hedgehog (see Protocol S1). Development of a mature *Drosophila* oocyte involves an elaborate sequence of events that must be precisely orchestrated in time. A surprising number of the genes in the above list play roles in important events that must take place within a specific window of time during oogenesis, many of which involve signaling between the germline and soma. Thus, an important emergent theme of miRNA regulation may revolve around the widespread need for precise control of spatiotemporally restricted events during development. In addition, oogenesis in *Drosophila* occurs through a very different developmental program than in vertebrates. It is thus intriguing that a single microRNA has potentially evolved to include a wide array of target genes that are important for this developmentally divergent process. However, many of these potential targets are not restricted to oogenesis but also function at other times and places, including the eye, nervous system, and epithelia, and a number of other predicted *miR-210* targets also function in these tissues (e.g., *arrowhead, cacophony, trio, Sema-1b, makorin, Van Gogh, Syntaxin 17, G-oα47A, RhoGAP92B, cul-2, Apc,* and *Scm*). Thus, this microRNA may play more complex pleiotropic roles in developmental networks. We conclude that some microRNAs could be candidates for genes that mediate clade-specific differences in gene expression, and could play an important role in shaping the diversity of life.

## Materials and Methods

### 

#### 3′ UTR alignments.

We used two sets of 3′ UTR alignments for flies. Set 1 was created on the basis of alignments, retrieved from the UCSC Genome Browser database at http://www.genome.ucsc.edu [30], by assembling aligned contigs of six fly species. The following assemblies were used to construct the* multiz* alignments [31]: *D. melanogaster* Apr. 2004 (dm2), *D. yakuba* Apr. 2004 (droYak1), *D. ananassae* Jul. 2004 (droAna1), *D. pseudoobscura* Aug. 2003 (dp2), *D. virilis* Jul. 2004 (droVir1), *D. mojavensis* Aug. 2004 (droMoj1), *Anopheles gambiae* Feb. 2003 (anoGam1), and *Apis mellifera* Jul. 2004 (apiMel1). The detailed amount of nucleotides and aligned sequence for all flies are shown in Tables 1 and 2. The 3′ UTR alignments of set 2 were extracted from genome-wide multiple alignments generated by the Pachter group at UC Berkeley (http://hanuman.math.berkeley.edu/genomes/drosophila.html) [21] using the following assemblies: *D. melanogaster* Apr. 2004 (dm2), *D. ananassae* Jul. 2004 (droAna1), *D. yakuba* Apr. 2004 (droYak1), *D. erecta* Oct. 2004, *D. pseudoobscura* Aug. 2003 (dp1), *D. virilis* Jul. 2004 (droVir1), *D. mojavensis* Dec. 2004. For both datasets we used FlyBase release 4.1 to extract 3′ UTRs in *D. melanogaster.*

#### microRNA sequences.

We downloaded all *D. melanogaster* microRNA precursors and mature microRNAs from the microRNA registry at Rfam [32] (release 5.0, http://www.sanger.ac.uk/Software/Rfam/mirna/index.shtml). For each microRNA, we checked for conservation of the precursor sequence in all fly species, using multiple alignments retrieved from the UCSC Genome database. We required the first 8mer of the mature microRNA to be perfectly conserved, but applied a less stringent conservation constraint, a percentage identity of 75%, to the precursor sequence. From the 79 mature *D. melanogaster* microRNAs, we found 69 to be conserved in all flies and 73 to be conserved in the *melanogaster* and *obscura* groups. Statistics were generated with a subset of 46 microRNAs with unique nuclei, i.e., each nucleus is specific for only one microRNA in this list. Lists of these microRNAs are provided as Tables S2–S4.

#### Randomized microRNAs.

Randomized microRNAs [13] were produced by extracting 8mers with the same genome-wide abundance (± 15%) in all *D. melanogaster* 3′ UTRs of the first and the second 7mer nucleus compared to the respective 7mers of the corresponding real microRNA. The 3′ end of the real microRNA was attached to this 8mer. We produced five cohorts of unique randomized microRNAs each for set 1 and set 2, in either case both with masked and unmasked repeats.

#### Different settings for PicTar predictions.

Comparing anchor site predictions based on the two different alignment sets (see Figure 2), we found that using alignment set 1 yielded an overall higher sensitivity, while target predictions based on set 2 had a higher specificity. A major determinant of sensitivity and specificity is the required level of conservation of anchor sites. According to these findings, we defined three PicTar settings (termed S1, S2, and S3) to cover the observed ranges of sensitivity and specificity. Masking repeats and applying free energy filtering of anchor sites served to fine-tune the trade-off between sensitivity and specificity for each setting. The high-sensitivity setting (S1) had repeat-masked UCSC alignments (set 1) as input sequences, required conservation of anchor sites only between species of the *melanogaster* and *obscura* groups, and applied no free energy filtering of perfect nuclei. Setting S2, providing high-specificity predictions, used alignments of set 2 with unmasked repeats as input sequences and required conservation of anchors in all flies and free energy filtering of perfect nuclei. The medium sensitivity/medium specificity setting S3 was equal to setting S1, but used conservation of anchors in all flies.

#### Phylogenetic PicTar score.

Given an alignment of a 3′ UTR for all flies, PicTar computes a likelihood score for the UTR of each species separately. The final score of the whole alignment is a weighted average of the single species scores, with weights reflecting the phylogenetic grouping of the species. More precisely, the score of all flies in the *melanogaster* subgroup was averaged and the resulting score was further averaged with the score for *D. ananassae* and *D. pseudoobscura,* yielding a score for the *melanogaster* and *obscura* groups. The scores for *D. mojavensis* and *D. virilis,* which have similar evolutionary distances to the *melanogaster* group, were averaged. This outgroup score and the score of the *melanogaster* and *obscura* groups were averaged to obtain the final PicTar score for all flies.

#### Homologous microRNAs between vertebrates and flies.

According to a recent study, the nucleus of a given microRNA is presumably sufficient to achieve repression of a gene [14]. We thus applied a relaxed definition of homology. Whenever the first or second 7mer of a microRNA in *Drosophila* was also present as one of the nuclei in a human microRNA, these two microRNAs were assumed to be homologs. Comparing all microRNAs conserved in the *melanogaster* and *obscura* groups with all microRNAs conserved in mammals, we obtained 48 pairs of homologous microRNAs between mammals and flies.

#### Target numbers for random microRNA pairs.

To assess the significance of targeting by 24 pairs of microRNAs extracted from clustered microRNA genes, we used 1,000 sets of 24 pairs of microRNAs drawn randomly from the set of all possible 1,128 distinct pairs (using all 48 unique microRNAs conserved in the *melanogaster* and *obscura* groups). For conservation of anchor sites in the *melanogaster* and *obscura* groups, on average 18 (± 2) out of 24 random pairs had at least one target gene, compared to 22 of the co-expressed pairs. We obtained on average 70 (± 21) unique target genes per random set, compared to 132 unique targets of the clustered pairs with a high *Z*-value (*Z* = 3). When requiring conservation between all flies, the results were more significant: 19 out of 24 clustered pairs targeted 50 unique genes, while on average 11 (± 2) out of 24 randomly drawn doublets were predicted to target approximately 23 (± 8) unique genes (*Z* = 3.5).

#### Homologous genes in vertebrates and flies.

Homologous genes between *D. melanogaster* and human were extracted from HomoloGene [33] (ftp://ftp.ncbi.nih.gov/pub/HomoloGene/current/) with annotations of 14 March 2005. This list contained 19,685 human genes and 7,983 fly genes. Keeping only pairs of homologous genes for which we were able to assign a FlyBase CG number and a RefSeq gene identifier [34], our reduced list contained 4,623 pairs of homologous genes. We extracted an additional list of homologous human–*D. melanogaster* transcripts from the Ensembl Genomebrowser (http://www.ensembl.org/). After merging both lists, we obtained a final list containing 8,136 pairs of homologous transcripts.

#### Shuffling test for homology relationships.

To asses the significance of the number of conserved microRNA–target relations of homologous target genes and microRNAs between vertebrates and flies, we shuffled homology relations in vertebrates and flies in the following way: All nonhomologous genes and microRNAs were discarded from our table of microRNA–target gene assignments. All microRNAs of a given family with equal 7mers at the 5′ end were represented by one specific member of this family. Similarly, we discarded multiple transcript variants, keeping only the longest variant for each gene. We constructed a list with assignments of each microRNA to all its target genes. Shuffling was performed by permuting the microRNA entries of this list, thereby assigning a new set of target genes to each microRNA. We counted the number of homology relationships for these permuted microRNA–target assignments and averaged the results over 1,000 runs. We obtained on average 45 (± 9) homology relationships for the shuffled lists, while we counted 60 real homology relationships, when using only unique lists of genes and microRNAs. The described shuffling strategy models a situation of nonconserved microRNA–target relations, but keeps the number of microRNAs targeting a particular gene constant.

#### GO term analysis.

To evaluate the PicTar target predictions for all single microRNAs, we searched for significantly overrepresented GO terms [20] of all target genes for each microRNA separately using the GeneMerge software [26]. GeneMerge computes the significance of occurrences of particular GO terms for a set of genes compared to a background gene set. To use an extensive background gene set that captures features of genes targeted by microRNAs as best possible, we lumped together all genes predicted to be targeted by all microRNAs (setting S1) or genes that were hit by the five cohorts of randomized microRNAs. Finally, *p*-values were conservatively corrected for multiple testing as provided by GeneMerge and recorded below a cutoff of 0.1. We performed the analysis separately for all GO terms in the “biological processes” ontology, and the most specific “biological processes” GO term for each gene, as well as for all GO terms in the “molecular function” ontology. These three classes of GO terms are provided by GeneMerge. Results from the first two analyses were merged into one output file, keeping the lower *p*-value for GO terms that were present twice. To visualize the results, we used two-way hierarchical clustering based on the linear correlation coefficient of the negative logarithm of the *p*-value [35]. To compute *p*-values for the overrepresentation of GO terms for genes that are (a) conserved between *D. melanogaster* and human, and (b) predicted to be targeted by homologous microRNAs in flies and mammals, we used a background gene set that was obtained by intersecting the background gene set described above with the set of all *D. melanogaster* genes with homologs in human.

## Supporting Information

Protocol S1 Detailed Discussion of Predicted *miR-210* Targets (170 KB DOC).

Table S1 Homologous Genes between Flies and Mammals, Targeted by Homologous microRNAs (71 KB XLS).

Table S2 Mature microRNAs Conserved in All Flies of Our Dataset (18 KB XLS).

Table S3 Mature microRNAs Conserved in the *melanogaster* and *obscura* Groups (20 KB XLS).

Table S4 Set of Unique, Conserved Mature microRNAs Used to Compute Signal-to-Noise Ratios (18 KB XLS).

## Abbreviation

GO: Gene Ontology
