# Supplementary material for: microRNA Target Predictions across Seven Drosophila Species and Comparison to Mammalian Targets
Source: PLoS Comput Biol. 2005 Jun 24;1(1):e13. doi: 10.1371/journal.pcbi.0010013 (PMC1183519; doi:10.1371/journal.pcbi.0010013)
Supplement: Protocol S1 — (170 KB DOC). [file pcbi.0010013.sd001.doc]

**Supplementary Discussion**

A significant proportion of predicted *miR-210* target genes in *Drosophila* carry the GO annotation “female gamete generation” and play well-characterized roles during oogenesis. A surprising number participate in important events that must take place within a specific window of time during oogenesis, many of which involve signaling between the germline and soma. Of particular interest, three genes (*Ras85D, grk, rho-4*) are directly involved in EGF RTK signaling, which is essential for establishing both the anterior-posterior (A-P) and dorso-ventral (D-V) axes of the future embryo and for follicle cell morphogenesis and migration (see [1] and references therein). Ras is a small GTPase that mediates RTK/MAPK signaling. Gurken is an EGF ligand that is required during mid-oogenesis to establish A-P polarity and later to establish D-V polarity in the oocyte [2], and is also the guidance cue for the RTK/Ras-dependent migration of border cells, a specialized population of follicle cells [3]. Gurken and other EGF ligands must be processed in the ER and Golgi in order to become activated, and this processing requires Rhomboid proteins [1, 4]. A fourth gene, *lozenge*, encodes a runt box transcription factor with similarity to human Acute Myeloid Leukemia 1 (AML1) that plays an important role in cell fate specification in eye development and hematopoesis (reviewed in [reviewed in 5, 6]). Some *lz* alleles result in female sterility due to defects in spermathecal development and sperm storage [7]. Combinatorial regulation by Lz in conjunction with EGFR and Notch signaling is necessary and sufficient to direct gene expression of specific target genes at the correct time during development [5].

Three predicted targets are implicated during oogenesis in processes that affect Notch and/or Wg/Hh signaling. Notch signaling plays critical roles in the specification and maintenance of the follicle cell epithelium [8], while Hh and Wg are important for embryonic patterning events that are dependent on D-V specification during oogenesis. The Cut homeodomain protein is a transcription factor that is both a target and effector of Notch and can also influence Wg signaling [9]. Cut functions as a determinant of cell-type specification in several tissues [9], and has also been implicated in cell cyle progression and control of nuclear matrix attachment regions [9]. During oogenesis, *cut* is implicated in the proper differentiation of interfollicular stalk cells [8] and is required for maintenance of egg chamber integrity, apparently through effects on soma-to-germline signalling [10]. Egghead is an integral membrane protein with beta-1,4-mannosyltransferase activity whose role in glycosphingolipid biosynthesis [11] is important to maintain adhesion between the follicle cell layer and oocyte by a mechanism thought to depend on Notch [12]. Mutations in *slalom*, which encodes a component of the secretory pathway that is required for sulfonation of glycosaminoglycans and is thought to affect processing of the D-V determinant *pipe*, cause Wg and Hh phenotypes [13]. Sll may affect multiple signaling pathways that depend on proteoglycans for efficient activation [13].

Other molecules implicated as potential targets of miR-210 (*Rbp9, par-1, gcl,* and *sn*) also play important roles during oogenesis. *Rbp9* encodes and RNA binding protein similar to *Drosophila* Elav and human Hu proteins, which are thought to regulate the stability of mRNAs that control cell proliferation/differentiation decisions (see [14]). Rbp9 protein is found in neuronal nuclei and the cytoplasm of ovaries and is required for proper sex determination of the female germline [15] as well as germline cystocyte differentiation and oocyte determination and positioning [14]. Rbp9 binds to the *bag-of-marbles* (*bam*) mRNA, which encodes a developmental regulator of germ cells that promotes the mitotic cystocyte divisions required to produce the 16-cell germline cyst [14]. Par-1 is a conserved Ser/Thr kinase that is important for cell polarity in a wide variety of cellular contexts [16]. Par-1 is required early during oogenesis for polarizing the germline cyst and for oocyte specification [17, 18], and later localizes to the posterior of the developing oocyte, where it is important for establishing A-P polarity and localization of posterior determinants [19, 20]. It is also required in the surrounding follicle cells for apical-basal polarity [21] and organization of polarized microtubules [22]. Mammalian Par-1 homologs are also implicated in neurodegenerative diseases such as Alzheimer’s through their ability to phosphorylate the microtubule-binding protein tau, which is a component of neurofibrillary tangles in amyloid plaques [23]. *germ cell-less* mRNA localizes to the posterior of the oocyte and is an essential component of the germ plasm, which is necessary for the specification of the germ cell lineage in the embryo; in early embryos, Gcl localizes to the nuclear membrane and represses transcription during establishment of the germ cell lineage, an activity that is essential for the formation of germ cell precursors (reviewed in [reviewed in 24]). Finally, *singed* encodes the actin-bundling protein fascin, which is required for the formation of actin filament bundles in developing bristles and nurse cells [25]. Female sterile *singed* alleles fail to assemble the cytoplasmic actin bundles that hold nurse cell nuclei in place during the rapid phase of cytoplasmic transfer, causing nurse cell nuclei to become lodged in ring canals (intercellular bridges between the nurse cells and the oocyte), thus preventing the transport of materials from the nurse cells into the developing oocyte and resulting in defective eggs [25].

Although these molecules perform a diverse set of molecular functions, the action of each is required in a transient fashion at specific times and locations during oogenesis and other developmental processes. This shared feature suggests that miRNA regulation could play a widespread role in spatio-temporal restriction of gene activity.

**References**

1. Shilo BZ (2003) Signaling by the *Drosophila* epidermal growth factor receptor pathway during development. Exp Cell Res 284: 140-9.

2. Gonzalez-Reyes A, Elliott H, and St Johnston D (1995) Polarization of both major body axes in *Drosophila* by *gurken-torpedo* signalling. Nature 375: 654-8.

3. Duchek P and Rorth P (2001) Guidance of cell migration by EGF receptor signaling during *Drosophila* oogenesis. Science 291: 131-3.

4. Urban S, Lee JR, and Freeman M (2002) A family of Rhomboid intramembrane proteases activates all *Drosophila* membrane-tethered EGF ligands. Embo J 21: 4277-86.

5. Canon J and Banerjee U (2000) Runt and Lozenge function in *Drosophila* development. Semin Cell Dev Biol 11: 327-36.

6. Coffman JA (2003) Runx transcription factors and the developmental balance between cell proliferation and differentiation. Cell Biol Int 27: 315-24.

7. Bloch Qazi MC, Heifetz Y, and Wolfner MF (2003) The developments between gametogenesis and fertilization: ovulation and female sperm storage in *Drosophila* *melanogaster*. Dev Biol 256: 195-211.

8. Dobens LL and Raftery LA (2000) Integration of epithelial patterning and morphogenesis in *Drosophila* ovarian follicle cells. Dev Dyn 218: 80-93.

9. Nepveu A (2001) Role of the multifunctional CDP/Cut/Cux homeodomain transcription factor in regulating differentiation, cell growth and development. Gene 270: 1-15.

10. Jackson SM and Blochlinger K (1997) *cut* interacts with *Notch* and protein kinase A to regulate egg chamber formation and to maintain germline cyst integrity during *Drosophila* oogenesis. Development 124: 3663-72.

11. Wandall HH, Pizette S, Pedersen JW, Eichert H, Levery SB, Mandel U, Cohen SM, and Clausen H (2005) Egghead and Brainiac are essential for glycosphingolipid biosynthesis in vivo. J Biol Chem 280: 4858-63.

12. Goode S, Melnick M, Chou TB, and Perrimon N (1996) The neurogenic genes *egghead* and *brainiac* define a novel signaling pathway essential for epithelial morphogenesis during *Drosophila* oogenesis. Development 122: 3863-79.

13. Luders F, Segawa H, Stein D, Selva EM, Perrimon N, Turco SJ, and Hacker U (2003) *slalom* encodes an adenosine 3'-phosphate 5'-phosphosulfate transporter essential for development in *Drosophila*. Embo J 22: 3635-44.

14. Kim-Ha J, Kim J, and Kim YJ (1999) Requirement of RBP9, a *Drosophila* Hu homolog, for regulation of cystocyte differentiation and oocyte determination during oogenesis. Mol Cell Biol 19: 2505-14.

15. Lee SH, Kim Y, and Kim-Ha J (2000) Requirement of Rbp9 in the maintenance of *Drosophila* germline sexual identity. FEBS Lett 465: 165-8.

16. Pellettieri J and Seydoux G (2002) Anterior-posterior polarity in *C. elegans* and *Drosophila*--PARallels and differences. Science 298: 1946-50.

17. Cox DN, Lu B, Sun TQ, Williams LT, and Jan YN (2001) *Drosophila par-1* is required for oocyte differentiation and microtubule organization. Curr Biol 11: 75-87.

18. Huynh JR, Shulman JM, Benton R, and St Johnston D (2001) PAR-1 is required for the maintenance of oocyte fate in *Drosophila*. Development 128: 1201-9.

19. Shulman JM, Benton R, and St Johnston D (2000) The *Drosophila* homolog of *C. elegans* PAR-1 organizes the oocyte cytoskeleton and directs *oskar* mRNA localization to the posterior pole. Cell 101: 377-88.

20. Tomancak P, Piano F, Riechmann V, Gunsalus KC, Kemphues KJ, and Ephrussi A (2000) A *Drosophila* melanogaster homologue of *Caenorhabditis elegans par-1* acts at an early step in embryonic-axis formation. Nat Cell Biol 2: 458-60.

21. Benton R and St Johnston D (2003) *Drosophila* PAR-1 and 14-3-3 inhibit Bazooka/PAR-3 to establish complementary cortical domains in polarized cells. Cell 115: 691-704.

22. Doerflinger H, Benton R, Shulman JM, and St Johnston D (2003) The role of PAR-1 in regulating the polarised microtubule cytoskeleton in the *Drosophila* follicular epithelium. Development 130: 3965-75.

23. Drewes G, Ebneth A, and Mandelkow EM (1998) MAPs, MARKs and microtubule dynamics. Trends Biochem Sci 23: 307-11.

24. Leatherman JL and Jongens TA (2003) Transcriptional silencing and translational control: key features of early germline development. Bioessays 25: 326-35.

25. Cant K, Knowles BA, Mooseker MS, and Cooley L (1994) Drosophila *singed*, a fascin homolog, is required for actin bundle formation during oogenesis and bristle extension. J Cell Biol 125: 369-80.
